# Nutrient profiling assessment of packaged snack foods with nutrition-related claims available on the Indian market

**DOI:** 10.3389/fnut.2024.1425354

**Published:** 2024-10-09

**Authors:** Amisha Bhatnagar, Monika Choudhary, Vikas Kumar, Vicky Singh, Prabhdeep Kaur

**Affiliations:** ^1^Department of Food and Nutrition, Punjab Agricultural University, Ludhiana, India; ^2^Department of Food Science and Technology, Punjab Agricultural University, Ludhiana, India; ^3^Department of Soil Science, Punjab Agricultural University, Ludhiana, India

**Keywords:** nutrient profiling, nutrient comparative claim, nutrient content claim, nutrition label, packaged snack foods

## Abstract

**Background:**

Food packaging includes labels with specific nutrient contents to provide consumers with nutritional information. Legislative actions and consumers’ growing interest in nutrition information have increased the disclosure of nutrition information. The study was planned to examine packaged snack foods carrying nutritional claims for nutrition labeling.

**Methods:**

The selected packaged chips were divided into categories based on the primary ingredients mentioned on the nutrition label, such as cereal/pseudocereal-based, millet-based, vegetable-based, and pulses/legume-based chips. Four threshold levels, such as total sugar, sodium, total fat, and saturated fat, were used for categorization.

**Results:**

Out of a total of 23 packaged chips, the corresponding 2, 7, 8, and 13 products had a higher content of sugar, saturated fat, sodium, and total fat than the threshold levels. A significant (*p* ≤ 0.01) difference was observed in the values of nutrients analyzed through laboratory methods in comparison with the values given on the nutrition label in the majority of the products.

**Conclusion:**

The majority of the products belonged to national brands and differed from the nutrition-related information given on the product label in terms of the nutrient content claim.

## 1 Introduction

Consumers prefer to consume flavorful and healthful food alternatives. Moreover, younger people prefer snacks, and India has one of the greatest proportions of younger people worldwide (1). Generally, processed foods enjoyed as snacks are those consumed in small-to-moderate portions between meals (1). These foods can be anything from highly processed, high-calorie products such as potato chips and baked products to less processed items such as nuts and dry fruits. The market has expanded due to the increased consumption of processed foods caused by sedentary lifestyles and demanding work schedules (2). Meanwhile, it has been anticipated that the worldwide snack market is expected to grow at a compound annual growth rate (CAGR) of 5.8% (1). In India, packaged snack food consumption continues to grow as a result of convenient choices, hygienic concerns, and rising purchasing power. This is evidenced by the expanding Indian Snacks Market, which is projected to grow at a compound annual growth rate (CAGR) of 13.2% between 2020 and 2026 (3). The market for snacks in India was valued at Rs 42,694.9 crore in 2023 and is expected to rise at a robust growth rate of 9.08 over the forecast period, reaching 95521.8 crore by 2032, out of which the overall market of salty snack foods in India is estimated to be worth Rs 2,500 crore. In just 13 years, the retail value of packaged junk food and soft drinks in India increased by 42.1 times, from US $0.9 billion in 2006 to over US $37.9 billion in 2019, and is still growing at a rapid rate (4).

Nutrient profiling methods (NPMs) are methodical techniques for grouping foods according to their ingredients, nutrient content, or other attributes (such as processing stages or essential versus optional meals). The underlying NPMs are crucial for front-of-package labeling systems because they determine whether goods are labeled with the warning label system and correctly classify products with high concentrations of nutrients of concern, such as sugar, salt, or saturated fat (5). Front-of-package labels (FOPLs) have two main objectives: to improve the nutritional quality of food purchases and to quickly and easily inform consumers about the nutritional quality of food. A secondary objective is to encourage reformulation in the food supply. In addition to giving consumers information on nutritional content, interpretive FOPLs are especially promising since they assist consumers in determining the healthfulness (or unhealthiness) of items and offer counsel (encouragement or discouragement) when making purchasing decisions (6).

When it comes to deterring consumers from buying foods high in nutrients of concern, warning labels may have the best evidence. Recent systematic assessments of experimental and quasi-experimental data have demonstrated that warnings can reduce the selection of unhealthy items by 26 to 36%. Recent research that concentrated on sugar discovered that the best way to increase consumers’ awareness of food’s high nutritional content was to utilize warnings. Evidence from Chile, the first nation to impose mandatory front-of-pack warnings, revealed that these labels assisted parents and kids in identifying harmful food and beverages and discouraging their consumption. They were also connected to a 24% drop in the purchase of unhealthy goods (7).

The World Health Organization (WHO) recommended in 2004 that one tactic to assist customers in choosing healthier food products is to provide them with nutrition labels. Food information, such as nutrition labeling, is more crucial than ever in packaged food products and aids customers in making knowledgeable decisions when buying packaged food items (8). A nutritional label is essentially a description meant to teach consumers about the nutritional qualities of food and help them choose foods that are likely to be balanced in terms of nutrients. The Ministry of Health and Family Welfare oversees the Food Safety and Standards (Packaging and Labeling) Regulations (2011) in India, which are carried out by the Food Safety and Standard Authority of India (FSSAI). The criteria for packaging and labeling packaged food products manufactured for commercial purposes are provided by this act to Indian food manufacturers, who must adhere to the specified guidelines (9).

Companies in the fast-moving consumer goods (FMCG) sector are aware that Gen-Z and Millennials represent significant consumers in the snack market. Majority of Indians encourage healthy snacks due to their features such as low calories, low fat, high fiber content, and protein-rich. Numerous leading Indian companies have created healthy but accessible snack products. The widespread view is that children’s consumption patterns, food preferences, and nutrition understanding are negatively affected by modern advertisements, which may eventually end up in harmful health outcomes such as obesity (10). Thus, nutrition-related claims may encourage customers, especially desk job doers and parents, to make healthier preferences. Any statement, suggestion, or inference that a food has specific nutritional qualities, such as energy value, protein, fat, carbohydrate content, or vitamin and mineral content, is referred to as a nutrition claim (11). However, the existence of a claim could make certain customers consider products with nutrition-related claims more favorably. It is critical to evaluate the nutritional composition of food to find out whether it is compliant. Therefore, the current research was undertaken to assess nutrient profiling of packaged snack foods available in the Indian market that make nutrition-related claims.

## 2 Materials and methods

The current study was carried out in Ludhiana City, Punjab. It is the most populous and the largest city in the Indian state of Punjab, with an estimated population of 1,618,879 as per the 2011 census. Ludhiana is also known as the industrial capital of the state, and it is home to several educational institutions and is a hub for business and trade.

Chips are marketed/advertised as a ‘cool’ munch, and they are also one of the most visible instant snack items among the fastest-moving packaged snacks at any grocery, supermarket, or department store. Therefore, the present study evaluated the nutrient profile of packaged chips.

### 2.1 Materials

The samples were procured from local supermarkets, discount, retail, and online stores. All sampled packaged chips were bought, and the packaging was retained. The experiments in this study were divided into two phases: categorization of packaged chips using the nutrient profile models (NPMs) and nutrient profiling of packaged chips for the evaluation of nutrient content claims.

### 2.2 Categorization of packaged chips using NPMs

The nutrients listed on the food labels of packaged chips with nutrition-related claims were considered. The samples were divided into categories based on the primary ingredients mentioned on the nutrition label, such as cereal/pseudocereal-based, millet-based, vegetable-based, and pulses/legume-based chips. The NPMs provided by the World Health Organization (WHO) were used to further categorize the selected packaged chips based on the threshold limits (12). Four threshold levels such as total sugar, sodium, total fat, and saturated fat were used for categorization (Table 1). The values of the above-mentioned components and total energy for percent energy calculations were taken from the product label.

**Table 1:** Threshold limits of sodium, total sugars, total fat, and saturated fat for the categorization of packaged snack foods.

Threshold level	Threshold limits	Interpretation
Sodium	If sodium: energy ≥1:1	High in sodium
Total sugar	If energy from free sugars ≥10% of total energy	Excessively high
Total fat	If energy from total fat ≥30% of total energy	Extremely high
Saturated fat	If energy from saturated fat ≥10% of total energy	Oversaturated

For sugar threshold levels, a product is “excessively high” in sugars, if the calories from free sugars [free sugars (g) x 4] are more than or equal to 10% of the product’s total energy. The following formula was used to determine the sugar threshold levels:


$$\mathrm{Sugar content}\;\left(g\right)\;x\;4=\mathrm{Calories from sugar}\;\left(X\right)$$

This value (X) should not be greater than 10% of total energy.

For sodium threshold levels, a product is considered high in sodium if the ratio of its sodium content (mg) to its energy content (Kcal) is above 1:1. The following formula was used to determine sodium threshold levels:


$$\frac{\mathrm{Sodium content}\;\mathrm{on}\;\mathrm{nutrition label}\;\left(\mathrm{mg}\right)}{\mathrm{Total energy}\;\mathrm{on}\;\mathrm{nutrition label}\;\left(\mathrm{Kcal}\right)}=X$$

This ratio (X) should not be greater than 1:1.

In terms of total fat threshold levels, a product is “extremely high” in total fats, if the calories from total fat [total fat (g) x 9] are more than or equal to 30% of the product’s total energy. The formula used to determine total fat threshold levels was:


$$\mathrm{Total}\;\mathrm{fat}\;\mathrm{content}\;\left(g\right)\;x\;9=\mathrm{Calories from total}\;\mathrm{fat}\;\left(X\right)$$

This value (X) should not be greater than 30% of total energy.

With regard to saturated fat threshold levels, a product is “oversaturated” in saturated fats, if the calories from saturated fat [saturated fat (g) x 9] are more than or equal to 10% of the product’s total energy. The following formula was used to determine saturated fat threshold levels:


$$\mathrm{Saturated}\;\mathrm{fat}\;\mathrm{content}\;\left(g\right)\;x\;9=\mathrm{Calories from saturated}\;\mathrm{fat}\;\left(X\right)$$

This value (X) should not be greater than 10% of total energy.

### 2.3 Type of nutrition-related claims present on packaged chips

The packaging of the selected packaged chips was examined for nutrition/health-related claims such as “protein rich,” “heart healthy,” “calcium rich,” and “good source of vitamins and minerals.” The number of claims was recorded and counted.

### 2.4 Nutrient content claims

The nutrients such as energy, carbohydrates, protein (13), fat (14), fatty acids (15), total sugars (16), fiber (13), dietary fiber (13), vitamin A (17), vitamin C (18), sodium, potassium (19), and iron and calcium (20) were determined in selected samples. By using the Kjeldahl method, crude protein (% total nitrogen x 6.25) was calculated. Fat was estimated by Soxhlet apparatus using 5 g of sample and petroleum ether as a solvent for extraction. Two grams of samples were burned for 5 h at 550° C in a muffle furnace to quantify the amount of ash present. After digesting 2 g of the fat-free sample with H2SO4 and NaOH, the residue was burned for 5 h at 550° C in a muffle furnace to produce crude fiber. Two grams of each sample were heated to a constant weight in a crucible inside an oven kept at 105° C to estimate the moisture content, and carbohydrate content was estimated by subtracting the total of all the proximates from 100 (100-moisture-crude protein-crude fat-crude fiber-ash).

For mineral content, the samples were first acid-digested and then further used for sodium, potassium, iron, and calcium estimation. A flame photometer was used for the estimation of sodium and potassium. AOAC (20) method was used for iron and calcium analyses. Estimation of vitamin C was conducted using standard procedures given in AOVC (18) (2,6-dichlorophenolindophenol dye solution method). For vitamin A analysis, hexane and acetone were used for the separation of the contents, and the reading was measured spectrophotometrically.

Fatty acid profiling was conducted by the formation of esters from the samples by the Appelqvist (15) method using the gas chromatography model. Total sugars were estimated using Dubois et al. (16) by Phenol sulphuric acid method. Using Megazyme-K-TDFR-200A, the samples’ soluble, insoluble, and total dietary fiber contents were examined in triplicate. The standard procedure provided by AOAC (13) was used to evaluate the amounts of soluble and insoluble dietary fibers.


$$\mathrm{Dietary fiber percentage}=\frac{\frac{R1+R2}{2}-p-A-B}{\frac{m1+m2}{2}}\times 100$$

where: R_1_ = residue weight 1 from m1, R_2_ = residue weight 2 from m_2_, m_1_ = sample weight 1, m_2_ = sample weight 2, A = ash weight from R_1_, p = protein weight from R_2_ and


$$B=\mathrm{blank}=\frac{BR1+BR2}{2}-BP-\mathrm{BA}$$

where: BR = blank residue, BP = blank protein from BR1, and BA = blank ash from BR2.

The nutrients present in selected packaged chips were evaluated using standard procedures, and the results of the laboratory analysis were compared to the values given on the nutrition label in accordance with the Food Safety Standard Authority of India (FSSAI) regulations or any particularly stated nutrient on the product label (11) to determine the complacency of the packaged chips with the nutrition-related claims.

### 2.5 Statistical analysis

All the determinations were carried out in triplicate, and the results are given in mean ± standard deviation. The Student’s *t*-test was applied to compare the data regarding nutrient analysis using Statistical Package for the Social Sciences (SPSS) 26 software, and the level of significance was set at a *p*-value of < 0.01 and *p*-value of < 0.05.

## 3 Results and discussion

To assess nutrient claims, a total of 23 packaged chips from 9 different brands were purchased for this study. The nutrients provided on food labels of packaged chips having nutrition-related claims were noted. The NPMs were implemented to categorize packaged chips.

### 3.1 Categorization of the selected products using NPMs

The four threshold levels for total sugar, sodium, total fat, and saturated fat established in NPMs were used to categorize the selected packaged chips (Table 2).

**Table 2:** Categorization of the selected packaged products using the nutrient profiling model.

Product name	Sugar threshold			Total fat threshold			Saturated fat threshold			Sodium threshold
	Sugar (g)	Calories^1^ (Kcal)	10% E	Total fat (g)	Calories^2^ (Kcal)	30% E	Saturated fat (g)	Calories^3^ (Kcal)	10% E	Sodium: energy
Barbeque beetroot chips (Brand 6)	36.9	148	47	17.7	160	139	–	–	–	–
Black *chana* chips (Brand 5)	6	24	21	–	–	–	–	–	–	–
Beetroot *masala* chips (Brand 1)	–	–	–	40.8	367	173	19.2	173	57.5	1.2:1
Popped potato chips (Brand 8)	–	–	–				–	–	–	2.2:1
7-grain protein snack (Cream and onion) (Brand 2)	–	–	–	23.5	212	144	–	–	–	1.7:1
7-grain protein snack (Peri-peri) (Brand 2)	–	–	–	22.6	203	143	–	–	–	1.7:1
Paprika popped chips (Brand 6)	–	–	–	–	–	–	–	–	–	1.2:1
Un-junked *tandoori* chips (Brand 7)	–	–	–	15.7	141	137	6.3	57	45.5	4.1:1
Un-junked chili pizza chips (Brand 7)	–	–	–				4.9	44	44.3	2.5:1
Soya chips (Brand 3)	–	–	–	16.8	151	131	5.9	113	45.1	2.6:1
Beetroot chips (Brand 5)	–	–	–	17.6	159	122	–	–	–	–
*Nachni* jalapeno chips (Brand 1)	–	–	–	32.7	294	159	15.4	139	53.1	–
Quinoa cheezopeno chips (Brand 1)	–	–	–	44.3	399	179	17	153	59	–
Oats chips (Brand 5)	–	–	–	62.3	239	155	–	–	–	–
Nutty chips (Brand 7)	–	–	–	28	252	153	–	–	–	–
Soya *pudina* chips (Brand 5)	–	–	–	26.4	274	153	–	–	–	–
*Moong* dal chips (Brand 5)	–	–	–	34.5	311	195	–	–	–	–
Purple sweet potato chips (Brand 9)	–	–	–	–	–	–	9.2	83	48.3	–

E: Energy (from product label), *Recommendations for sugar threshold level: calories from sugar (Kcal) ≤ 10% of total energy, **total fat threshold level: calories from total fat ≤ 30% of total energy, ^#^saturated fat threshold level: calories from saturated fat (Kcal) ≤ 10% of total energy, ^##^sodium threshold level: sodium (mg): energy (Kcal) ≤ 1:1, ^1^ = sugar X4, ^2^ = total fat X 9, ^3^ = saturated fat X 9.

The analysis of sugar threshold levels of packaged chips revealed that barbeque beetroot chips (brand 6) had the highest sugar threshold level (148 Kcal). The black *chana* chips (brand 5) had 24 Kcal calories from sugar. Out of 23 products, 2 products had total sugar content higher than the suggested threshold limit. Out of the total, eight products had sodium content higher than the recommended threshold limit. According to the nutrition label, quinoa cheezopeno chips (brand 1) had the highest total fat threshold level, delivering 399 Kcal from total fat, followed by beetroot *masala* chips (brand 1), which provided 367 Kcal. A total of 13 products had total fat content above the threshold limit. The saturated fat threshold values for the beetroot *masala* chips, quinoa cheezopeno chips, and *nachni* jalapeno chips from brand 1 were 173, 153, and 139 Kcal, respectively. Among all, seven products showed saturated fat content above the permissible threshold limit.

### 3.2 Type of claims present on packaged chips

In this section of the study, the selected products were categorized as per the primary food ingredients mentioned on the nutrition label, such as cereal/pseudocereal-based chips, millet-based chips, vegetable-based chips, and pulses/legume-based chips. The selected products had 2–10 nutrient claims, while the range of health claims was 2–7.

In the present study, food products under the cereal/pseudocereal-based chip category exhibited 2–5 claims such as “protein rich,” “fiber rich,” “baked, not fried,” and “gluten free.” Millet-based chips had 3–6 types of claims such as “improves digestion,” “iron rich,” “cholesterol free,” and “low glycemic index (GI).” The nutrition label of vegetable-based chips showed 4–10 claims such as “vacuum fried,” “boosts energy levels,” “50 percent less oil than regular chips,” and “vegan friendly.” The pulses/legume-based chip category had 3–6 claims such as “low calorie,” “high content of complex carbohydrates and fibre,” and “rich in vitamin A.”

### 3.3 Nutrient content claims

The present research reported that the energy, total fat, dietary fiber, and sodium contents of 70, 57, 80, and 75% of the cereal/pseudocereal-based chips were significantly (*p* ≤ 0.01) lower than the values listed on the nutrition label (Table 3). In 28% of the products examined in the laboratory, higher protein levels were observed. The sugar content of 50% of the products that underwent laboratory examination was significantly (*p* ≤ 0.01) less than what was stated on the nutrition label. In comparison with the values stated on the nutrition label, the calorie, carbohydrate, and saturated fatty acid (SFA) contents of 66, 50, and 33% of the millet-based chips were significantly (*p* ≤ 0.01) higher (Table 4). Furthermore, a high level of protein was found in 33% of the products tested in the laboratory. In total, 50 and 83% of the millet-based chips had lower total fat and dietary fiber contents, respectively, than the values listed on the nutrition label. Fifty percent of the products that were subjected to laboratory analysis had significantly (*p* ≤ 0.01) lower monounsaturated fatty acid (MUFA) and polyunsaturated fatty acid (PUFA) contents than the amounts indicated on the nutrition label.

**Table 3:** Nutrient profiling of packaged cereal/pseudocereal-based chips.

Product name	Nutrients	Nutrition label on packaging	Laboratory analysis	*p*-value	*T*-value	CI	
						Lower	Upper
Quinoa cheezopeno chips (Brand 1)	Energy (Kcal)	596	466 ± 1.6	< 0.001**	−139.46	−193.77	−40.37
	Protein (g)	8.6	11 ± 0.6	0.002**	7.554	1.77	10.55
	Total fat (g)	44.3	22.2 ± 0.3	< 0.001**	−135.81	−185.10	−38.56
	SFA (g)	17	9.1 ± 0.3	< 0.001**	46.82	13.24	63.65
	MUFAs (g)	15.6	9.9 ± 0.2	< 0.001**	108.60	30.83	148.02
	PUFAs (g)	10.6	3.1 ± 0.03	< 0.001**	321.32	91.29	437.90
	Carbohydrates (g)	40.6	55.3 ± 1	< 0.001**	27.43	7.68	37.45
	Dietary fiber (g)	10.6	0.2 ± 0	< 0.001**	4470.57	6089.90	1270.27
	Sugar (g)	2.1	1.6 ± 0.06	< 0.001**	−12.63	−17.37	−3.35
	Calcium (mg)	148	87 ± 0.3	< 0.001**	−399.33	−544.22	−113.46
	Iron (mg)	3.1	2.8 ± 0	< 0.001**	−69.21	−94.36	−19.62
	Potassium (mg)	440	498.6 ± 2.3	< 0.001**	45.03	12.73	61.42
	Sodium (mg)	585	420.7 ± 1.1	< 0.001**	−248.66	−338.89	−70.64
	Vitamin C (mg)	4.4	1.9 ± 1.5	< 0.001**	17.01	4.663	23.30
	Vitamin A (μg)	65	12.8 ± 0.2	< 0.001**	−448.29	−610.93	−127.37
Oats chips (Brand 5)	Energy (Kcal)	517	515 ± 1.1	0.044*	−2.90	−4.55	−0.05
	Protein (g)	7.1	5.6 ± 0.15	< 0.001**	−17.45	−23.9	−4.79
	Total fat (g)	26.6	27.3 ± 0.2	0.002**	7.69	1.82	10.74
	Carbohydrate (g)	62.3	61.8 ± 0.3	0.016*	−3.97	−5.87	−0.50
	Sugar (g)	9.8	1.3 ± 0.04	< 0.001**	−313.11	−426.71	−88.95
Brown rice *biryani* chips (Brand 5)	Energy (Kcal)	2010	533 ± 0.3	< 0.001**	7438.17	0	2113.26
	Total fat (g)	22.3	29.9 ± 0.2	< 0.001**	81.88	23.22	111.61
	Carbohydrate (g)	60.7	61 ± 0.3	0.139^NS^	1.84	−0.44	3.33
	Fiber (g)	4.5	0	0	0	0	0
Paprika popped chips (Brand 6)	Energy (Kcal)	423	415 ± 1.5	< 0.001**	−9.15	−12.68	−2.28
	Protein (g)	7.9	1.4 ± 0.04	< 0.001**	−238.27	−324.72	−67.69
	Total fat (g)	9.2	8.6 ± 0.2	0.004**	−6.11	−8.65	−1.29
	SFA (g)	0.5	1.4 ± 0.01	<0.001**	120.90	34.32	164.77
	Carbohydrate (g)	67.5	83.1 ± 0.2	< 0.001**	124.45	35.33	169.62
	Sugar (g)	0.6	1.1 ± 0.1	< 0.001**	9.77	2.48	13.51
	Sodium (mg)	524.3	593.5 ± 2.5	< 0.001**	48.90	13.83	66.69
Nutty chips (Brand 7)	Energy (Kcal)	510	491 ± 4.1	0.001**	−7.85	−10.95	−1.87
	Protein (g)	15	16 ± 0.1	< 0.001**	15.76	4.29	21.60
	Total fat (g)	28	24.2 ± 0.2	< 0.001**	−35.84	−48.89	−10.10
	SFA (g)	2	2.2 ± 0.03	< 0.001**	199.48	56.66	271.86
	Carbohydrate (g)	50	52.3 ± 1.5	0.054^NS^	2.69	−0.03	4.31
	Dietary fiber (g)	7	9.3 ± 0	< 0.001**	513.83	145.99	700.26
	Sugar (g)	2.3	2.2 ± 0.05	0.051^NS^	2.75	−4.37	0.01
Un-junked *tandoori* chips (Brand 7)	Energy (Kcal)	455	483 ± 1.9	< 0.001**	26.13	7.31	35.69
	Protein (g)	12	9.4 ± 0.1	< 0.001**	−35.91	−49.00	−10.12
	Total fat (g)	15.7	22.5 ± 0.5	< 0.001**	23.48	6.54	32.09
	SFA (g)	6.3	2.4 ± 0.1	< 0.001**	14.53	3.92	19.93
	Carbohydrate (g)	66.7	70 ± 0.7	< 0.001**	14.82	−20.33	−4.01
	Dietary fiber (g)	6.9	2.7 ± 0	< 0.001**	−1550.94	−2113.61	−440.68
	Sugar (g)	6.9	16.7 ± 0.5	< 0.001**	34.51	9.72	47.09
	Sodium (mg)	1866.9	668.7 ± 2.3	< 0.001**	−892.99	−1216.97	−253.73
Un-junked chili pizza chips (Brand 7)	Energy (Kcal)	443	493 ± 2	< 0.001**	42.93	12.13	58.55
	Protein (g)	14.3	9.1 ± 0.07	< 0.001**	−114.03	−155.42	−32.37
	Total fat (g)	12	22.4 ± 0.4	< 0.001**	47.98	13.57	65.43
	SFA (g)	4.9	1.8 ± 0	< 0.001**	483.5	137.37	658.91
	Carbohydrate (g)	69.6	63.7 ± 0.5	< 0.001**	−20.35	−27.83	−5.63
	Dietary fiber (g)	8.7	2.5 ± 0.03	< 0.001**	−290.21	−395.50	−82.45
	Sugar (g)	6.8	23.8 ± 0.5	< 0.001**	64.64	18.32	88.12
	Sodium (mg)	1093.6	601.1 ± 2	< 0.001**	−422.46	−575.73	−120.03

Values are mean ± SD (*n* = 3), saturated fatty acids (SFAs), monounsaturated fatty acids (MUFAs), polyunsaturated fatty acids (PUFAs), **significant at 1% level of significance, *significant at 5% level of significance, NS—no significant difference.

**Table 4:** Nutrient profiling of packaged millet-based chips.

Product name	Nutrients	Nutrition label on packaging	Laboratory analysis	*p*-value	*t*-value	CI	
						Lower	Upper
*Nachni* jalapeno chips (Brand 1)	Energy (Kcal)	531	490 ± 1.4	< 0.001**	−51.91	−70.79	−14.69
	Protein (g)	5.7	8.6 ± 0.6	0.001**	7.82	1.86	10.91
	Total fat (g)	32.7	25.1 ± 0.1	< 0.001**	−83.55	−113.89	−23.70
	SFA (g)	15.4	11.6 ± 0.2	< 0.001**	120.79	34.29	164.62
	MUFAs (g)	13.3	10.6 ± 0.1	< 0.001**	125.21	35.55	170.65
	PUFAs (g)	3.8	2.9 ± 0.1	< 0.001**	25.77	7.20	35.20
	Carbohydrates (g)	53.4	57.3 ± 0.6	< 0.001**	12.00	3.16	16.52
	Dietary fiber (g)	7	0.3 ± 0.01	< 0.001**	−855.92	−1166.45	−243.20
	Sugar (g)	0.9	4.2 ± 0.1	< 0.001**	88.95	25.24	121.24
	Calcium (mg)	183	156.8 ± 0.6	< 0.001**	−75.18	−102.49	−21.32
	Iron (mg)	2.5	2.8 ± 0.04	< 0.001**	9.78	2.48	13.53
	Potassium (mg)	305	429.5 ± 1.4	< 0.001**	150.37	42.70	204.94
	Sodium (mg)	420	570 ± 2.4	< 0.001**	106.96	30.36	145.78
	Vitamin C (mg)	2.4	3 ± 2	< 0.001**	23.96	6.68	32.74
	Vitamin A (μg)	40	29.3 ± 0.5	< 0.001**	−34.28	−46.77	−9.65
7-grain protein snack (Cream and onion) (Brand 2)	Energy (Kcal)	481	485 ± 1.5	0.007**	5.06	0.91	7.28
	Protein (g)	16.6	15.4 ± 0.3	0.004**	−5.97	−8.46	−1.24
	Total fat (g)	23.5	22.6 ± 0.1	< 0.001**	−13.28	−18.24	−3.55
	SFA (g)	4.6	5.2 ± 0.2	< 0.005**	33.44	9.41	45.63
	MUFAs (g)	10.1	10 ± 0.1	< 0.016*	503.30	143	685.9
	PUFAs (g)	8.6	7.5 ± 0.03	< 0.001**	977.82	277.83	1332.57
	Carbohydrate (g)	49	55.1 ± 0.4	< 0.001**	26.14	7.31	35.7
	Dietary fiber (g)	6.6	1.9 ± 0.01	< 0.001**	−500.84	−682.55	−142.30
	Sugar (g)	3.1	1.8 ± 0.06	< 0.001**	−34.50	−47.07	−9.71
	Sodium (mg)	833	780.5 ± 1.7	< 0.001**	−77.68	−105.9	−22.03
7-grain protein snack (Peri-peri) (Brand 2)	Energy (Kcal)	477	453 ± 1	< 0.001**	−41.15	−56.14	−11.62
	Protein (g)	16.6	15.8 ± 0.05	< 0.001**	−22.96	−31.38	−6.39
	Total fat (g)	22.6	17.9 ± 0.1	< 0.001**	−52.79	−71.98	−14.94
	SFA (g)	4.5	4.6 ± 0.05	< 0.117^NS^	68.88	19.53	93.90
	MUFAs (g)	9.6	7.4 ± 0.06	< 0.001**	2444.38	694.55	3331.20
	PUFAs (g)	8.3	5.8 ± 0.1	< 0.001**	129.21	36.69	176.11
	Carbohydrate (g)	50	57.2 ± 0.3	< 0.001**	39.81	11.23	54.31
	Dietary fiber (g)	6.6	1.9 ± 0.01	< 0.001**	−500.84	−682.55	−142.3
	Sugar (g)	3	1.9 ± 0.01	< 0.001**	−103.76	−141.42	−29.45
	Sodium (mg)	833	1082.2 ± 1.8	< 0.001**	238.30	67.7	324.77
*Ragi* chips (Brand 4)	Energy (Kcal)	424	477 ± 2.9	< 0.001**	31.91	8.97	43.55
	Protein (g)	6	7.4 ± 0.06	< 0.001**	40.15	11.33	54.76
	Total fat (g)	9	23.7 ± 0.4	< 0.001**	69.68	19.75	95
	Carbohydrate (g)	78	58.6 ± 0.6	< 0.001**	−52.92	−72.16	−14.98
Spiced *ragi* chips (Brand 6)	Energy (Kcal)	424	484 ± 1.1	< 0.001**	95.71	27.16	130.46
	Protein (g)	6	0.6 ± 0.04	< 0.001**	−187.48	−255.50	−53.25
	Total fat (g)	10	22.8 ± 0.3	< 0.001**	70.96	20.12	96.73
	Carbohydrate (g)	78	69.1 ± 0.5	< 0.001**	−31.98	−43.64	−8.99
	Dietary fiber (g)	7	0.06 ± 0	< 0.001**	−2971.85	−4050.03	−844.42
Barley millet chips (Brand 5)	Energy (Kcal)	355	511 ± 0.7	< 0.001**	399.84	113.6	544.9
	Protein (g)	12	5.2 ± 0.3	< 0.001**	−38.43	−52.43	−10.84
	Total fat (g)	3	26 ± 0.1	< 0.001**	282.14	80.15	384.51
	Carbohydrate (g)	74	63.6 ± 0.6	< 0.001**	−29.83	−40.72	−8.37
	Fiber (g)	17	0.02 ± 0	< 0.001**	−3059.36	−4169.29	−869.29

Values are mean ± SD (*n* = 3), saturated fatty acids (SFAs), monounsaturated fatty acids (MUFAs), polyunsaturated fatty acids (PUFAs), **significant at 1% level of significance, *significant at 5% level of significance, NS—no significant difference.

In vegetable-based chips, 60% of the laboratory analyzed products had significantly (*p* ≤ 0.01) higher SFA contents than the values listed on the nutrition label. Forty percent of the products examined in the laboratory showed significantly (*p* ≤ 0.01) higher protein contents (Table 5). Compared to the figures given on the nutrition label, 40 and 60% of the products had significantly (*p* ≤ 0.01) lower total fat and dietary fiber contents. Eighty percent of all the products evaluated in the laboratory had significantly (*p* ≤ 0.01) lower sugar content than the values displayed on the nutrition label. Among minerals, 20% of the products had significantly (*p* ≤ 0.01) higher sodium and potassium contents in laboratory analysis than the figures indicated on the nutrition label. Furthermore, the laboratory investigation revealed that 80 and 20% of the chips made from pulses/legumes had significantly (*p* ≤ 0.01) higher calorie and protein contents than the figures listed on the nutrition label (Table 6). Compared to the nutrition labels of the other four products in this category, the total fat content of just one laboratory-examined product was significantly (*p* ≤ 0.01) lower. Out of all the products examined in the laboratory, 60% of products had significantly (*p* ≤ 0.01) less carbohydrates.

**Table 5:** Nutrient profiling of packaged vegetable-based chips.

Product name	Nutrients	Nutrition label on packaging	Laboratory analysis	*p*-value	*t*-value	CI	
						Lower	Upper
Beetroot *masala* chips (Brand 1)	Energy (Kcal)	575	486 ± 1.9	< 0.001**	−82.01	−111.78	−23.26
	Protein (g)	7.1	3.1 ± 0.6	< 0.001**	14.86	4.02	20.39
	Total fat (g)	40.8	24.6 ± 0.1	< 0.001**	−187.25	−255.2	−53.191
	SFA (g)	19.2	10.4 ± 0.2	< 0.001**	59.55	16.87	81.2
	MUFAs (g)	16.5	10.7 ± 0.03	< 0.001**	302.12	85.83	411.73
	PUFAs (g)	4.6	3.2 ± 0.9	< 0.001**	93.81	26.62	127.87
	Carbohydrates (g)	44.8	53.9 ± 0.5	< 0.001**	30.84	8.67	42.1
	Dietary fiber (g)	4.5	0.5 ± 0.02	< 0.001**	−270.09	−368.09	−76.73
	Sugar (g)	7	4.6 ± 0.2	< 0.001**	−18.37	−25.144	−5.06
	Calcium (mg)	80	76.3 ± 0.3	< 0.001**	−25.69	−35.09	−7.18
	Iron (mg)	2	2.9 ± 0.04	< 0.001**	29.46	8.27	40.22
	Potassium (mg)	400	541 ± 1.7	< 0.001**	143.57	40.77	195.67
	Sodium (mg)	695	485.6 ± 1.5	< 0.001**	−244.78	−333.60	−69.54
	Vitamin C (mg)	2.6	2 ± 0.9	< 0.001**	35.28	9.94	48.14
	Vitamin A (μg)	45	2.3 ± 0.1	< 0.001**	−631.15	−860.13	−179.33
Beetroot chips (Brand 5)	Energy (Kcal)	408	502 ± 0.6	< 0.001**	268.76	76.35	366.27
	Protein (g)	4.8	5.5 ± 0.4	0.51^NS^	2.76	−0.007	4.38
	Total fat (g)	17.6	26.1 ± 0.1	< 0.001**	177.27	50.35	241.59
	Carbohydrate (g)	56	61.5 ± 0.4	< 0.001**	22.24	6.18	30.4
	Dietary fiber (g)	25.4	0.4 ± 0.0	< 0.001**	−7203.88	0	−2046.9
Barbeque beetroot chips (Brand 6)	Energy (Kcal)	464	353 ± 2.2	< 0.001**	−86.95	−118.52	−24.67
	Protein (g)	12.5	9.8 ± 0.3	< 0.001**	−18.27	−25.01	−5.03
	Total fat (g)	17.7	32.2 ± 0.02	< 0.001**	868.37	246.73	1183.42
	Carbohydrate (g)	63.7	6.1 ± 0.6	< 0.001**	−166.29	−226.64	−47.23
	Sugar (g)	36.9	23.7 ± 0.4	< 0.001**	−53.40	−72.81	−15.11
Popped potato chips (Brand 8)	Energy (Kcal)	440	432 ± 0.5	< 0.001**	−29.95	−40.88	−8.41
	Protein (g)	7.2	0.4 ± 0.02	< 0.001**	−534.36	−728.24	−151.83
	Total fat (g)	14	12.1 ± 0.1	< 0.001**	−24.93	−34.06	−6.96
	SFA (g)	3.2	2.8 ± 0.05	< 0.001**	136.19	38.67	185.62
	Carbohydrate (g)	67.2	80.4 ± 0.2	< 0.001**	105.46	29.93	143.74
	Sugar (g)	2.4	2.0 ± 0.05	< 0.001**	−12.72	−17.49	−3.38
	Sodium (mg)	961.2	377 ± 1.9	< 0.001**	−523.84	−713.90	−148.84
Purple sweet potato chips (Brand 9)	Energy (Kcal)	483	490 ± 0.9	< 0.001**	12.33	3.27	16.97
	Protein (g)	2.16	3.1 ± 0.1	< 0.001**	15.41	4.19	21.13
	Total fat (g)	12.8	22.7 ± 0.2	< 0.001**	84.58	23.99	115.29
	SFA (g)	9.2	10.1 ± 0.1	< 0.001**	149.77	42.53	204.13
	Unsaturated fat (g)	3.6	12.6 ± 0.1	< 0.001**	1226.27	348.43	1671.16
	Carbohydrate (g)	78	68.2 ± 0.2	< 0.001**	−96.26	−131.21	−27.32
	Dietary fiber (g)	17.5	12.7 ± 0.05	< 0.001**	−149.94	−204.35	−42.58
	Sugar (g)	6	16.1 ± 0.2	< 0.001**	89.97	25.53	122.64
	Sodium (mg)	211	535 ± 1.7	< 0.001**	319.51	90.77	435.43

Values are mean ± SD (*n* = 3), saturated fatty acids (SFAs), monounsaturated fatty acids (MUFAs), polyunsaturated fatty acids (PUFAs), **significant at 1% level of significance, NS—no significant difference.

**Table 6:** Nutrient profiling of packaged pulses/legume-based chips.

Product name	Nutrients	Nutrition label on packaging	Laboratory analysis	*p*-value	*t*-value	CI	
						Lower	Upper
Soya chips (Brand 3)	Energy (Kcal)	436	502 ± 0.4	< 0.001**	294.48	83.66	401.33
	Protein (g)	9.6	6.1 ± 0.1	< 0.001**	−42.28	−57.67	−11.94
	Total fat (g)	16.8	22.6 ± 0.04	< 0.001**	222.38	63.17	303.07
	SFA (g)	5.9	5.2 ± 0.1	< 0.001**	66.91	18.96	91.22
	MUFAs (g)	5.6	10.5 ± 0.04	< 0.001**	353.67	100.48	481.99
	PUFAs (g)	2.2	6.9 ± 0.05	< 0.001**	168.23	47.78	229.28
	Carbohydrate (g)	64.1	68.4 ± 0.1	< 0.001**	54.05	15.30	73.70
	Fiber (g)	4.5	0.1 ± 0.1	< 0.001**	−752.54	−1025.56	−213.82
	Sugar (g)	4	1.9 ± 0.02	< 0.001**	−164.46	−224.14	−46.71
	Calcium (mg)	147.1	162.2 ± 0.4	< 0.001**	69.18	19.61	94.31
	Iron (mg)	4.2	2.5 ± 0.01	< 0.001**	−211.54	−288.29	−60.09
	Sodium (mg)	1,131	390.3 ± 2.5	< 0.001**	−517.65	−705.46	−147.08
	Vitamin C (mg)	42.1	17 ± 1.7	< 0.001**	26.15	−35.72	−7.32
Black *chana* chips (Brand 5)	Energy (Kcal)	210	527 ± 1.3	< 0.001**	408.24	115.99	556.36
	Protein (g)	10.7	0.2 ± 0.02	< 0.001**	−770.38	−1049.87	−218.89
	Total fat (g)	3.8	32.1 ± 0.2	< 0.001**	288.68	82.01	393.42
	Carbohydrate (g)	35	59.3 ± 0.3	< 0.001**	168.30	47.80	229.38
	Sugar (g)	6	1.4 ± 0.05	< 0.001**	−147.55	−201.09	−41.90
Soya *pudin*a chips (Brand 5)	Energy (Kcal)	510	522 ± 1.7	< 0.001**	11.99	3.16	16.50
	Protein (g)	7.9	8.9 ± 0.2	< 0.001**	9.63	2.44	13.33
	Total fat (g)	26.4	28.1 ± 0.2	< 0.001**	11.99	3.16	16.51
	Carbohydrate (g)	60.1	58.2 ± 0.07	< 0.001**	−45.88	−62.57	−12.97
	Sugar (g)	1.56	1.1 ± 0.01	< 0.001**	−52.45	−71.52	−14.85
*Chatpata rajma* chips (Brand 5)	Energy (Kcal)	340	471 ± 0.8	< 0.001**	298.35	84.76	406.60
	Protein (g)	25	9.9 ± 0.1	< 0.001**	−196.49	−267.79	−55.81
	Total fat (g)	2	21 ± 0.07	< 0.001**	452.58	128.59	616.78
	Carbohydrate (g)	61	60.7 ± 0.07	< 0.001**	−6.96	−9.77	−1.58
	Sugar (g)	2.2	1.1 ± 0.1	< 0.001**	−15.73	−21.56	−4.28
*Moong dal* chips (Brand 5)	Energy (Kcal)	650	501 ± 0.1	< 0.001**	−2064.17	−2813.05	−586.51
	Protein (g)	24.8	12.9 ± 0.2	< 0.001**	100.46	136.93	−28.51
	Total fat (g)	34.5	25.3 ± 0.04	< 0.001**	−339.91	−463.23	−96.57
	Carbohydrate (g)	60.7	55.2 ± 0.3	< 0.001**	−30.15	−41.15	−8.47
	Fiber (g)	8.3	0.1 ± 0	< 0.001**	−1464.68	−1996.06	−416.17

Values are mean ± SD (*n* = 3), saturated fatty acids (SFAs), monounsaturated fatty acids (MUFAs), polyunsaturated fatty acids (PUFAs), **significant at 1% level of significance.

## 4 Discussion

This study provides an overview of the nutritional profiles of packaged snack foods that are sold in India and makes claims about their nutrition. Nutrient claims in particular can help consumers make confident decisions when choosing any kind of food. Any statement, suggestion, or inference that a food has specific nutritional qualities, such as energy value, protein, fat, carbohydrate content, or vitamin and mineral content, is referred to as a “nutrient claim” (21).

In this study, the nutrients listed on the labels of bagged chips that made claims about their nutrition were observed and were further categorized into four threshold levels as per NPMs. As per the WHO’s NPMs, a product is referred to be too high in sodium if its sodium-to-energy ratio is greater than 1:1 (12). In an earlier study, the nutrition labels of 5,008 pre-packaged food products showed that 44% of the products had excessive sodium levels (22). Similarly, out of 10,487 Canadian packaged food goods from 60 subcategories, food products from 59 categories had higher sugar content according to the nutrition label than those with the Front-of-Pack (FOP) mark (23). Moreover, Rincon-Gallardo Patino et al. (24) found that 2,544 food and non-alcoholic beverage commercials were broadcast for 275 food goods. Further a study examined 899 packaged food products, as per the nutrition label, 318 products (35%) were high in saturated fats (25).

Any representation that asserts, indicates, or suggests that a food possesses specific nutritional qualities, including but not limited to the energy value, the quantity of protein, fat, and carbs, as well as the presence of vitamins and minerals, is referred to as a nutrient claim (11). These claims may include information regarding vitamins, minerals, fiber, protein, or certain dietary components such as low fat, decreased sugar, high fiber, or an excellent source of a specific nutrient. One previous study reported that out of 2,034 packaged food and drinks, 295 had health claims, 64% had nutrition claims, and 6% had ingredient claims related to health (26). Earlier studies reported that fat-related claims (18.3%) and vitamin/mineral-related claims (15.4%) made up the largest percentage of nutrition content claims, followed by claims about calcium (11.5%) and sugar (11%) (27). In a study conducted by Duran et al. (28) from a total of 11,434 packaged foods and drinks, environmental claims, nutrition claims, and health claims were depicted in 5.2, 28.5, and 22.1% of food products, respectively. Furthermore, Pongutta et al. (29) collected data from 7,205 food products belonging to five major classes. Out of those, 1,487 products (20.6%) carried claims related to health. Similarly, Mayhew et al. (30) found that 737 packaged food products had two marketing methods and one health or nutrition claim on average. Among all, nutrition labels were found on 86% of packaged food products, followed by nutrition or health claims on 30% and specific marketing strategies on 87% of the products.

Nutrition-related claims may help parents, desk job doers, and other consumers to make healthier choices. Any representation that asserts, indicates, or suggests that food possesses specific nutritional qualities, including but not limited to the energy value, the quantity of protein, fat, and carbohydrate, as well as the presence of vitamins and minerals, is referred to as a nutrition claim (11). In contrast, one recent study examined 1,153 food products from 14 stores and documented that items with health or nutritional claims had significantly (*p* ≤ 0.01) lower levels of sugar (9.67 g/100 g), fat (9.2 g/100 g), saturated fat (3.2 g/100 g), and salt (371.36 mg/100 g) (31). Similarly, a study conducted in 2016 in Sydney, Australia, claimed that 34% of the products with nutrient claims did not meet their nutrient profiling criteria (32). Martinez-Perez and Arroyo-Izaga (33) examined the nutritional profile of 3,894 packaged food products and found that 48.6% of the products met the poor nutritional quality (LNQ) classification. Another research executed in Brazil from April to July 2017 examined 775 packaged food products made of grains. The findings indicated that the claims of “whole grains” were made by 19% of the evaluated products; of these, 35% did not include any whole grains among the top three components (32). Earlier, a study stated that from a total of 156 food products evaluated for children, 62.2% (*n* = 97) met the Food Standards Australia New Zealand nutrient profile assessment criteria, making them “less healthy.” The alternative core food grouping approach yielded a classification of “less healthy” for 66.7% (*n* = 104) of the products surveyed (34).

Consumers may be misled by claims believing that these goods are healthier than those without them, even if they may contain more of one or more essential nutrients.

### 4.1 Limitations and future research

The nutrient profiling model for the categorization of packaged snack foods in the context of the Indian population needs to be framed. Data on the consumption of packaged foods were limited in the context of India. So, further studies need to be conducted to assess the daily consumption of packaged snack foods with and without nutrition/health-related claims. Since wealthier people tend to allocate their food expenditure differently than poorer people, another promising way would be to analyze how trends and levels of processed food purchases differ across socioeconomic classes and other demographic characteristics. Finally, it is critical to look into the sociodemographic traits, purchasing patterns, and potential connections to dietary and health outcomes of subpopulations of regular packaged and processed food users.

## 5 Conclusion

The present research concluded that all the selected products belonged to one international and eight national brands. All the products had one of the nutrients, such as sugar, fat, sodium, or saturated fat, above the threshold limits. In the majority of the products, nutrient content analyzed through laboratory methods differed from the values given on the nutrition label. So, there is a need to frame a nutrient profiling model for the categorization of packaged snack foods in the context of the Indian population with the purpose of monitoring healthy packaged snack foods available in the market.

## Data Availability

The original contributions presented in the study are included in the article/supplementary material, further inquiries can be directed to the corresponding author.
